# Scanning Tunneling Spectroscope Use in Electrocatalysis Testing

**DOI:** 10.3390/ma3063675

**Published:** 2010-06-14

**Authors:** Turid Knutsen

**Affiliations:** Faculty of Engineering and Science, University of Agder, NO-4898 Grimstad, Norway; E-Mail: turid.knutsen@uia.no; Tel.: +47-37-253-065; Fax: +47-37-253-001

**Keywords:** scanning tunneling microscope, oxygen evolution reaction, metallic glasses of Ni alloys

## Abstract

The relationship between the electrocatalytic properties of an electrode and its ability to transfer electrons between the electrode and a metallic tip in a scanning tunneling microscope (STM) is investigated. The alkaline oxygen evolution reaction (OER) was used as a test reaction with four different metallic glasses, Ni_78_Si_8_B_14_, Ni_70_Mo_20_Si_5_B_5_, Ni_58_Co_20_Si_10_B_12_, and Ni_25_Co_50_Si_15_B_10_, as electrodes. The electrocatalytic properties of the electrodes were determined. The electrode surfaces were then investigated with an STM. A clear relationship between the catalytic activity of an electrode toward the OER and its tunneling characteristics was found. The use of a scanning tunneling spectroscope (STS) in electrocatalytic testing may increase the efficiency of the optimization of electrochemical processes.

## 1. Introduction

The search for the best catalyst in an electrochemical reaction is a time consuming and expensive process. A quick test for excluding unfitted materials, and for narrowing the list of promising catalysts to be selected for further electrochemical investigations, is the wish of all electrochemical researchers. Most electrochemical reactions begin with the adsorption of an ion to an active site of the uppermost surface of the catalyst, followed by an electron transfer between the adsorbed ion and the surface site of the catalyst. The transfer of electrons to or from the active site is, for many electrochemical reactions, the rate determining step. Finding an easy method of determining, roughly, a surface site’s ability to transfer electrons may therefore introduce a procedure for the selection of materials for further electrochemical investigations. The aim of this study was to find a relationship between the electrocatalytic properties of a catalyst and its ability to transfer electrons between two phases. In order to find such a relation, different nickel alloys were used as catalysts for the oxygen evolution reaction (OER) in alkaline water electrolysis. The catalytic properties of the alloys toward the OER were studied by electrochemical methods and then compared with the alloy’s surface’s ability to donate and accept electrons. This property was investigated by the means of a scanning tunneling microscope (STM). A quantity called the charge ratio (r_c_), obtained from I (U) curves given in the scanning tunneling spectroscope (STS) scans, was introduced. The quantity was used for the comparison of the shape of the curves within different samples.

The results of this work indicate that there exists a relationship between the electrocatalytic properties of the tested electrodes and their ability for tunneling electrons in the STM. An investigation of the tunneling properties of an electrocatalyst may therefore give an indication of the efficiency toward a given electrochemical reaction. The use of the STM in electrocatalyst testing may aid in the search for the optimal catalyst in many electrochemical processes.

## 2. Theory

The underlying physical basis of STM is electron tunneling. A detailed description of the tunneling current between the tip and the sample surface is given elsewhere [1], and an approximation for the total tunneling current, under the limit of low temperature and weak tip-sample interaction, can be written as:

(1) $$I\left(U\right)=I_{0}\cdot \int_{E_{f}}^{E_{f}+eU}n_{T}\left(E-eU\right)\cdot n_{S}\left(E\right)\cdot T\left(E,eU\right)dE$$

where Io = (2e/h) (e.s.); e.s. stands for an appropriate energy scale, nT and nS are the number of (dimensionless) local density of states at the given energy, for the tip and the sample, respectively, U is the applied bias voltage, E is the energy measured with respect to the Fermi level of the sample, and T (E, eU) is the tunneling transmission probability.

According to the quantum mechanical Wentzel, Kramer, Brillouin (WKB) approximation [2], the tunneling transmission probability for a one-dimensional trapezoidal barrier is given as:

(2) $$T\left(E,eU\right)=e^{-2s\;\cdot \;\sqrt{\frac{2m}{\hbar^{2}}\left(\Phi +\frac{eU}{2}-E\right)}}$$

Here *Φ* is the tunneling barrier potential, which as an approximation, is set to be the average of the working function of the tip and the sample, in units of e.s., *s* is the distance between the sample and the tip (in Å), and e.s. is one eV.

In ordinary STS measurements, the tip is placed over a point of interest on the sample surface, and the distance between the tip and the surface is fixed by momentarily interrupting the feedback controller. By scanning the applied voltage at a constant rate over a desired interval, while simultaneously measuring the tunneling current, a detailed dependence of the tunneling current on the applied voltage can be found. The recorded tunneling current corresponds to the electronic tunneling properties of the specific site on the surface, and provides information on the density of surface states and on the distribution of the applied field [3,4,5]. For STS investigations, the bias voltage, at which the feedback loops are interrupted, determined the distance between the tip and the sample. Hipps [6] gives a description of the similarity between an electron transfer in an electrochemical reaction and the tunneling process in the STS in his chapter about Orbital Mediated Tunneling Spectroscopy (OMTS) in the Handbook of Applied Solid State Spectroscopy.

**Figure 1:**
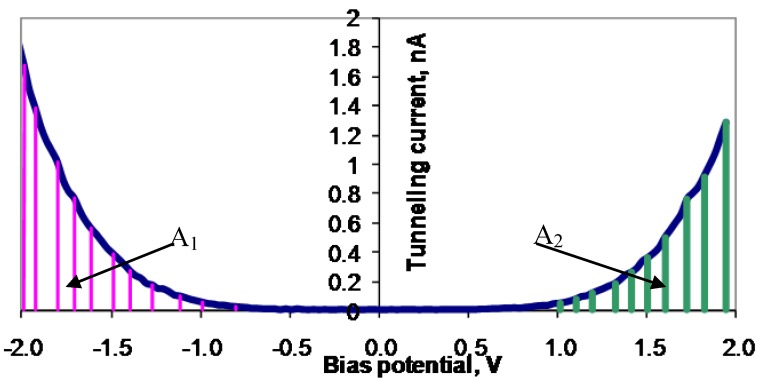
An example of an I(U) curve in STS. The absolute value of the tunneling current is used. The marked areas A_1_ and A_2_ are proportional with the charge amount transferred from sample to tip and from tip to sample, respectively. Scan rate: 0.002 s/V.

A typical example of an I(U) curve from an STS scan is illustrated in Figure 1. There are several notable features in an I(U) curve produced by an STS scan. The Fermi level of the sample is defined as the zero bias voltage. At a positive bias voltage, the rise in tunneling current normally indicates the bias potential where the Fermi level of the metallic tip matches energetically with the conduction band edge of the surface site of the sample. At a negative bias voltage, the rise in tunneling current occurs when the Fermi level of the tip matches the valence band edge of the surface sites. For some surface sites, there is a region around zero bias voltage where the junction capacitance is small, and hence the charging energy is big, and no apparent charge transport occurs. This situation is called the Coulomb blockage phenomena [7], and is seen in the I(U) curve as a flat region around the zero bias voltage, where no tunneling current takes place. Coulomb blockage phenomena are mainly observed on surfaces with semi-conductive properties. For a doped semiconductor, the Fermi level is moved away from the middle of the band gap. For an n-type conductor, there exist electron levels that are close to the conduction band, but with lower energies. The rise in the tunneling current will therefore have started at a negative bias voltage that was close to the zero bias voltage. For a p-type conductor, there are empty energy levels close to the valence band that lead to a rise in the tunneling current at positive bias voltages that are close to the zero bias voltage. For net n- or p-type doped semiconductors, the Coulomb blockage gap will be displaced, compared to the zero bias voltage. In an STS-scan, the size of the Coulomb blockage phenomena, and the position of the Fermi level, depends on whether the surface is a net n- or p-type semiconductor. For a pure metallic surface, there is no Coulomb blockage, and the Fermi level is placed at the uppermost top of the valence band.

### 2.1. The Charge Ratio

The shape of an I(U) curve depends on the mutual ability for a sample and a tip to accept and donate electrons. Due to the exponential dependence between the gap distance and the tunneling current (Equation 1), small differences in the distance involve large changes in the tunneling current. A direct comparison of the tunneling current in the I(U) scans performed on the surfaces of different samples will therefore involve large uncertainty. The normalized differential conductance (dI/dU)/(I/U) for metals and narrow-band gap semiconductors is often used for the study of electronic properties like the LDOS of the surface of a sample. However, according to Bando *et al.* [8], this method fails both at the ends of surface energy gaps and in the limit where |U| << Φ.

In order to compare the shape of the I(U) curves, with respect to the total ability of accepting or donating electrons, a quantity called the charge ratio is introduced. The charge ratio, r_c_, is based on the assumption that with a constant scan rate for the ramping voltage, the areas between the I(U) curve and the U axis are proportional to the total charge transfer between the tip and the sample. This is analogous to the calculation of charge transfer of an I(t) graph in electrochemical investigations. A feasible way to compare the I(U) curves for different samples will therefore be via the comparison of the charges transferred between the tip and the samples. The unit, r_c_, is defined as the ratio between the amount of charge tunneling from the occupied states of the sample surface to the tip (U < 0), q_occupied,_ and the amount of charge tunneling from the tip to the unoccupied states of the sample surface (U > 0), q_unoccupied_ (Equation 3). With a constant scan rate, the amount of charge is proportional with the size of the area between the curve and the potential axis. Thus, the amount of charge is easy to calculate by integration. Figure 1 gives an illustration of these areas.

(3) $$r_{c}=\frac{q_{occupied}}{q_{unoccupied}}=\frac{A_{1}}{A_{2}}$$

Here r_c_ is the charge ratio, A_1_ and A_2_ are the areas under the I(U), q_occupied_ is the total charge transfer from sample to tip when the bias voltage is scanned from −U to 0, and q_unoccupied_ is the total charge transfer from tip to sample when the bias voltage is scanned from 0 to U.

Considering the size of the charge ratio, one can conclude that if r_c_ > 1, the amount of electrons transferred from the sample surface to the tip is larger than the amount of electrons the sample surface may accept from the tip. The sample would therefore have more electrons in the conduction band, and less electron holes in the valence band, compared to the tip. Whilst for r_c_ < 1, the sample surface would have, in this case, a larger capacity to accept electrons from the tip, than to transfer electrons from the sample surface to the tip. The sample would have more electron holes in the valence band and less electrons in the conduction band compared to the tip. When the measured site is dominated by positively charged ions, it is reasonable to expect a higher ability to accept electrons from, rather than donate to, the tip, thus making r_c_ < 1. A negatively charge cluster will, of similar reason, give rise to r_c_ > 1. If the I(U) scans are performed by the same tip, differences in r_c_ indicate differences in the electronic properties of the surface sites.

### 2.2. A Theoretical Explanation of the Charge Ratio

For a theoretical description of the charge ratio, the expression for the tunneling current given in Equation 1 is used. If the bias voltage is scanned in the region [−U, U], the area can be calculated by A_1_ = $-\int_{-eU}^{0}I(U)du$ and A_2_ = $\int_{0}^{eU}I(U)du$. In order to calculate these integrals, expressions, for both the number of local densities of states (LDOS) at the given energy for the tip and the sample and for the tunneling transmission probability, have to be found. As an approximation, the tunneling potential barrier height, Φ, is considered constant, and the approximation for the tunneling matrix given in Equation 2 is used. The feedback interruption keeps the gap distance constant during the STS scans. The Fermi level of the sample is used as the zero level. As an approximation, the Fermi function at room temperature for both the tip and the sample is set to be unity, and the electrons are considered to be in ground state. The Equation 1 can be rewritten as:

(4) $$I(eU)=C\int_{0}^{eU}e^{-2s\cdot \frac{{\left(2m\right)}^{1/2}}{\hbar }\sqrt{\Phi +\frac{eU}{2}-E}}dE$$

The tunneling current can be calculated by as:

(5) $$I(eU)=C_{1}\left[\left(\sqrt{\left(\Phi +\frac{eU}{2}\right)}-1\right)e^{-2s\frac{\sqrt{2m}}{\hbar }\sqrt{\left(\Phi +\frac{eU}{2}\right)}}-\left(\sqrt{\left(\Phi -\frac{eU}{2}\right)}-1\right)e^{-2s\frac{\sqrt{2m}}{\hbar }\sqrt{\left(\Phi -\frac{eU}{2}\right)}}\right]$$

The charge ratio is found to be:

(6) $$r_{c}=\frac{q_{occupied}}{q_{unoccupied}}=\frac{\int_{-eU}^{0}I(eU)deU}{\int_{0}^{eU}I(eU)deU}$$

According to Onipko *et al.* [9], the LDOS at a flat metal surface is nearly constant at different places on the surface. With the approximation that the LDOS for the sample and tip are constant in the bias potential region, the charge ratio r_c_ will be equal to 1, and the scanning curve will be symmetric around the Fermi energy. With the first approximation, the r_c_ = 1 for electrons tunneling between two metal surfaces seems to be correct; the approximation corresponds with the statement of Magnov *et al.* [3] that the I(U) curve for a metal surface is symmetric around the Fermi level.

Unlike a flat metal surface, where the LDOS is set to be a constant value, the real shape of the STM tip is never known. It is, however, far from being ideally flat. Therefore, the approximation of a constant value for the LDOS of the tip is obviously not applicable. According to Bockris and Khan [10], an LDOS having an energy E (filled or not) is given per unit volume by Equation 7.

(7) $$\rho (E)=\frac{1}{2\pi^{2}}{\left(\frac{2m_{e}}{\hbar^{2}}\right)}^{3/2}E^{1/2}$$

Using this expression in Equation 1, the tunneling current can be written as:

(8) $$I(eU)=D\int_{0}^{eU}|E-eU{|}^{1/2}e^{-2s\cdot \frac{{\left(2m\right)}^{1/2}}{\hbar }\sqrt{\Phi +\frac{eU}{2}-E}}dE$$

Here D is a constant, including π, m_e_, and ħ.

Jurczyszyn and Stankiewicz [11] calculated the electronic structure of the sample surface by using the self-consistent, local consistent atomic orbital (LCAO) method. The LCAO Hamiltonian contains two contributions—the one-electron part, Ĥ^oe^, and the many-body part, Ĥ^mb^. They calculated the electronic structure by using the wave functions of the independent atoms that form the considered system. The many-body contributions were included by introducing the Hartree and exchange-correlation potentials for each orbital.

**Figure 2:**
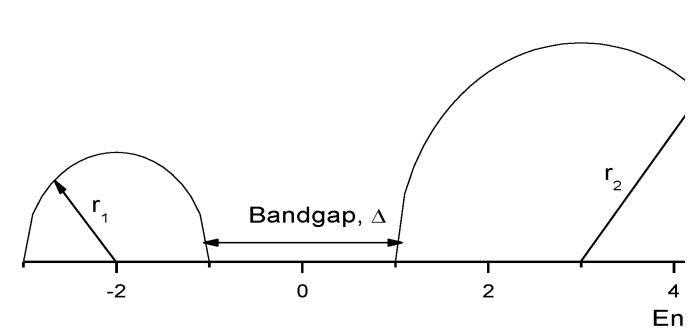
Schematic drawing of the semicircular band used in the calculation of the sample DOS showing the band gap ∆ around the Fermi level, E_F_.

According to Onipko *et al.* [9], the highest occupied molecular orbital (HOMO) and the lowest unoccupied molecular orbital (LUMO) are associated with the π electron states of molecules. It is assumed that oxide and hydroxide layers on metal surfaces react in the same way. Onipko showed, by calculations, that the molecular π electronic subsystem played a major role in formation of the tunnel current between the sample surface and the STM tip. For different types of oxides, Kar *et al.* [12] have worked out a model for the DOS, where they used the approximation of semicircular bands to ease the calculations of the DOS, as indicated by the sketch in Figure 2.

According to Kar *et al.* [12], a detailed shape of the bands is not as important in the energy range close to the band gap; the bandwidth of both the valence and the conduction band is much larger than the gap. The following relations can be used with Fermi energy as the zero of the energy for the sample DOS:

(9) $$n_{S}(E)={\left[r_{1}^{2}-{\left(E+r_{1}+\frac{\Delta }{2}\right)}^{2}\right]}^{1/2}\text{ for }E<-\frac{\Delta }{2}$$

(10) $$n_{S}(E)={\left[r_{2}^{2}-{\left(E-r_{2}-\frac{\Delta }{2}\right)}^{2}\right]}^{1/2}\text{ for }E>\frac{\Delta }{2}$$

(11) $$n_{S}(E)=0\text{ for }E\geq -\frac{\Delta }{2}\text{ and }E\leq \frac{\Delta }{2}$$

With these approximations for the LDOS of the sample and the expression for the LDOS of the tip given by Bockris and Khan [10], the equation for the tunneling current will then be given in Equations 12, 13 and 14.

(12) $$I(eU)=D\int_{0}^{-eU}{\left[r_{1}^{2}-{\left(E+r_{1}+\frac{\Delta }{2}\right)}^{2}\right]}^{1/2}{\left|E-eu\right|}^{1/2}\cdot e^{-2s\cdot \frac{{\left(2m\right)}^{1/2}}{\hbar }\sqrt{\Phi +\frac{eU}{2}-E}}dE\text{ for E}<-\frac{\Delta }{2}$$

(13) $$I(eU)=D\int_{0}^{eU}{\left[r_{2}^{2}-{\left(E-r_{2}-\frac{\Delta }{2}\right)}^{2}\right]}^{1/2}\cdot {\left|E-eU\right|}^{1/2}\cdot e^{-2s\cdot \frac{{\left(2m\right)}^{1/2}}{\hbar }\sqrt{\Phi +\frac{eU}{2}-E}}dE\text{ for E}>\frac{\Delta }{2}$$

(14) $$I(eU)=0\text{ for E}\geq -\frac{\Delta }{2}\text{ and E}\leq \frac{\Delta }{2}$$

Equation 14 can explain the Coulomb blockage gap around zero bias voltage in the I(U) curves observed for semiconductors. According to this approximation, the gap will correspond to the energy gap between the LUMO and the HOMO of the molecules, or the oxides, on the sample surfaces.

The r_c_ may then be calculated by the expression:

(15) $$r_{c}=\frac{\int_{-A}^{-\Delta /2}D\int_{0}^{-eU}{\left[r_{1}^{2}-{\left(E+r_{1}+\frac{\Delta }{2}\right)}^{2}\right]}^{1/2}{\left|E-eu\right|}^{1/2}\cdot e^{-2s\cdot \frac{{\left(2m\right)}^{1/2}}{\hbar }\sqrt{\Phi +\frac{eU}{2}-E}}dE\;]dU}{\int_{\Delta /2}^{A}[D\int_{0}^{eU}{\left[r_{2}^{2}-{\left(E-r_{2}-\frac{\Delta }{2}\right)}^{2}\right]}^{1/2}\cdot {\left|E-eU\right|}^{1/2}\cdot e^{-2s\cdot \frac{{\left(2m\right)}^{1/2}}{\hbar }\sqrt{\Phi +\frac{eU}{2}-E}}dE]dU}$$

To describe the STM tip more exactly, the cluster-Bethe-lattice method can be used. In this approach, the topmost part of the tip is represented by a pyramidal cluster of five atoms, with a single atom located at the apex of the pyramid, and four atoms forming its base. The atoms of the base are joined to Bethe-lattices, which simulate the influence of the rest of the tip. The LDOS of the STM tip will be a mixture of states, with s, p, and d type symmetry. According to Hofer and Redinger [13], the best agreements between experiments and simulations were achieved by setting the electronic structure of the tip as a mixture of states with s and d_z_2 type symmetry.

Jurczyszyn and Stankiewicz [11] found that the size of the tunneling current between the tip and the sample does not solely depend on the energy and shape of the orbital directly involved in the tunneling process. The size was also modified by the destructive or constructive interferences of inter-atomic orbitals (especially s-p_z_ interferences). In the surfaces of different Ni-alloys, the alloying elements, oxides, and hydroxides may interfere with the atomic orbitals of the Ni-atoms, resulting in a modification of the tunneling current.

A simulation for the charge ratio, where the LDOS for both the sample and the tip are expressed by solving the Schrödinger equation, where the wave functions are expressed by a summation of the atomic orbitals for both the samples and the tip, may be done. By using either a Hartree-Fock or a Density Functional calculation, an expression for the wave functions may be found for both the tip and the sample surface. For further reading about the theory of STM, the work of Blanco *et al.* [14] is recommended.

## 3. Experimental

The nickel alloys used in these investigations were the metallic glasses Ni_78_Si_8_B_14,_ Ni_70_Mo_20_Si_5_B_5_, Ni_58_Co_20_Si_10_B_12_, and Ni_25_Co_50_Si_15_B_10_. All four alloys were delivered by Vakuumschmelze GmbH, Hanau, Germany. The materials were produced by rapid quenching, from the melt, on a spinning casting wheel, with a cooling rate higher than 10^6^ K/s [15]. They were delivered as ribbons with a width of *ca.* 10 mm and a thickness of 20–50 μm. The EDS investigations were performed with a FEI Quanta 200 FEG-EDEM.

Images of the surfaces were found by scanning tunneling microscope (STM) and the electronic properties of the surfaces were studied by scanning tunneling spectroscopy (STS). The STM and STS measurements were conducted *ex situ* with a Jeol JSPM-4210 at atmospheric pressure and room temperature and 80% Pt-Ir tips, which were sharpened by anodic etching in KCN solution. The specimens were mounted on the STM-holder with Ag-paste. The polarity of the bias voltage was defined in the conventional way, *i.e.*, for negative sample bias voltage, electrons tunnel from the sample to the STM tip. In the STS, the I(U) data were collected in the spectroscopy mode with the feedback loop turned off. The I(U) data represented an average of 128 consecutive voltage sweeps of ± 2.0 V collected at five sites situated at a 30 × 30 nm^2^ topographic image. All repeated at three randomly chosen regions on the surfaces. The charge ratios were calculated and used as tools in the comparison of the shapes of the I(U) curves. All the STM investigations were performed in ambient atmosphere and the hydroxide film was not removed.

The electrochemical experiments were performed using a Gamry CMS 100 potentiostat. Cyclic voltammograms between hydrogen and oxygen evolution were made in a 1 M KOH electrolyte-solution at 25 °C. The KOH solution was made from a Dilute-it analytical concentrate, delivered from J.T. Baker, and distilled water. In order to remove oxygen, N_2_ was purged through the cell and the measurements were made under an Nitrogen atmosphere without bubbling. A three electrode setup was used. A double-walled glass cell with a water bath (Haake K Fisons) provided thermostatic control of the electrolyte. The counter electrode was composed of Ni-plates with large surfaces. All the electrodes were treated in an ultrasonic bath of ethanol for 15 min. and rinsed in distilled water. The electrodes were mounted in a metal clip and the area exposed to the electrolyte was measured. A saturated calomel electrode was used as the reference electrode. Cyclic voltammograms were run between −0.8 and 0.8 V_SCE_. The scan rate used was 5 mV/s. The potential scans were repeated until steady I = f(U) contours were attained (3–4 cycles).

The steady state current density at the overpotential of 0.6 V was found after 150 min. of polarization. The overpotential values were obtained by the relation $\eta =E-E_{O_{2}/OH^{-}}^{o}$, where E is the applied potential and $E_{O_{2}/OH^{-}}^{o}$ is the theoretical equilibrium Nernst potential in 1 M KOH at 25 ○C ($E_{O_{2}/OH^{-}}$ = 0.157 V_SCE_). According to Nikolov *et al.* [16], the Tafel slopes at high current density can be affected by the factors encountered in studying gas evolution on porous electrodes: blockage of the electrochemically active sites by gas bubbles, thus reducing the surface area available for an electrochemical reaction. The Tafel slopes at high current density will therefore not be further discussed in this study. The Tafel slopes at low current density were calculated by linear approximation in the direction of decreasing potential in order to reduce the effect of the oxidation of Ni on the surface.

## 4. Results

### 4.1. Electrochemical Results

EDS analyses were performed on the surfaces where B was not detected due to the small weight of the atom. The results are listed in Table 1. Taking into account the lack of B in the measured content of the different species of the alloys, the measurements were in agreement with the nominal contents.

**Table 1:** EDS analysis of the different metallic glasses with Ni.

Sample	Atom % Ni	Atom % Si	Atom % Mo	Atom % Co
Ni_78_Si_8_B_14_	90 ± 2	10 ± 2		
Ni_70_Mo_20_Si_5_B_5_	74	6	20	
Ni_58_Co_20_Si_10_B_12_	65	11		24
Ni_25_Co_50_Si_15_B_10_	30	18		52

**Figure 3:**
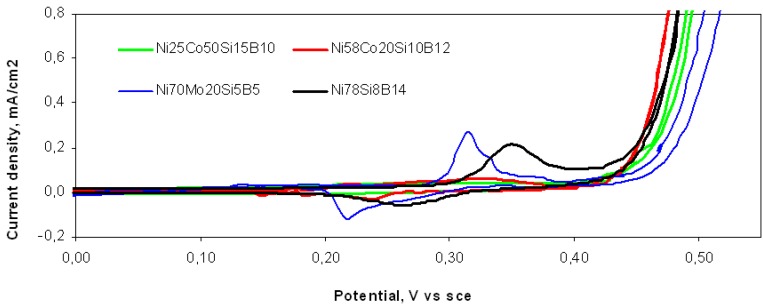
Cyclic voltammogram curves for four different Ni-containing metallic glasses in 1 M KOH at 25 ^○^C in N_2_ atmosphere. Scan rate: 5.0 mV/s.

**Figure 4:**
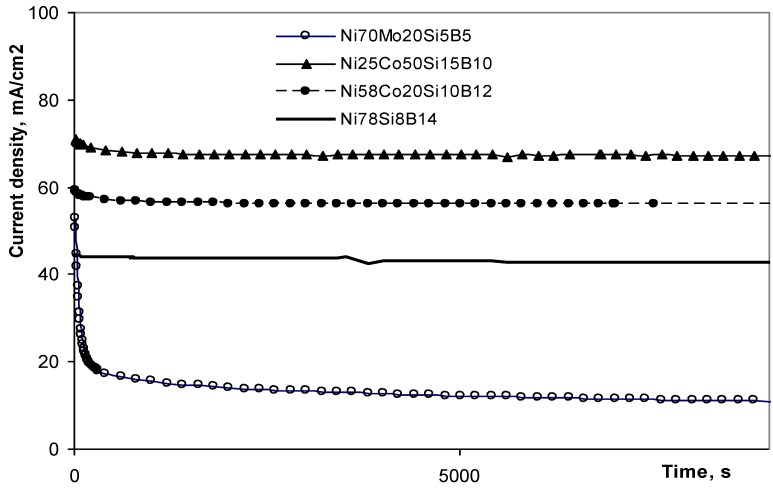
Polarization curves for four different Ni-containing metallic glasses in 1 M KOH at 25 ^○^C in N_2_ atmosphere at an overpotential of 0.6 V toward the OER.

**Figure 5:**
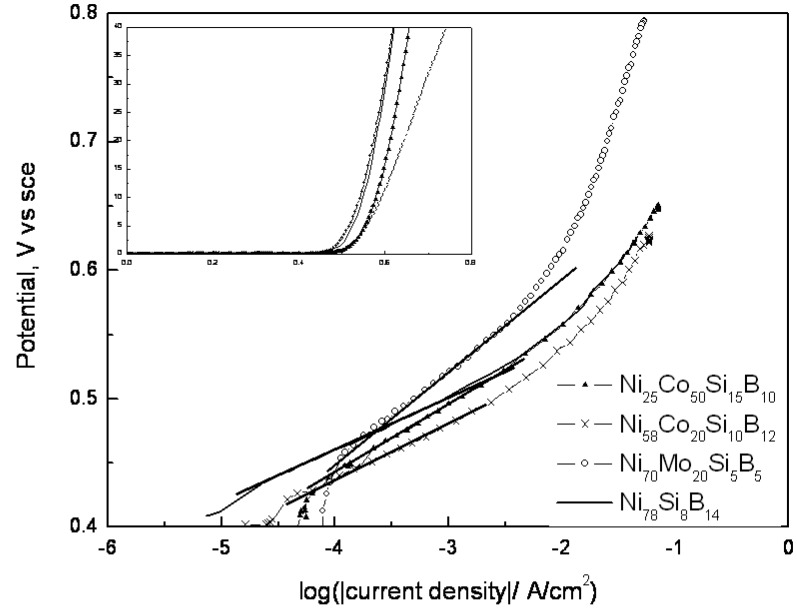
Tafel curves for Ni containing metallic glasses in 1 M KOH at 25 ^○^C in the N_2_ atmosphere. The inserted CV-Figure reveals that the Tafel slopes at high potentials change with the alloys.

The cyclic voltammograms are shown in Figure 3, the polarization curves in Figure 4, and the Tafel curves in Figure 5. Both the peak positions and the areas for the Ni(OH)_2_/NiOOH redox reaction changed with the elemental contents of the alloys investigated. The voltammograms for Ni_58_Co_20_Si_10_B_12_ and Ni_25_Co_50_Si_15_B_10_ exhibited broad anodic peaks corresponding to the oxidation of Co(II)/Co(III), α-Ni(II)/Ni(III), β-Ni(II)/Ni(III), and Co(III)/Co(IV) [16]. Both anodic peaks had a cathodic shift compared to that of Ni_78_Si_8_B_14_. Ni_78_Si_8_B_14_ and Ni_70_Mo_20_Si_5_B_5_ both had one anodic peak corresponding to Ni(II)/Ni(III) at 0.350 and 0.320 V_sce_, respectively. The sharp rise in the current density, due to the OER, starts approximately between 0.4 and 0.45 V_sce_ for all four alloys.

**Table 2:** Electrochemical data of the OER for the different metallic glasses with Ni.

Sample	Tafel slope mV/dec	Current density at η = 0.6V mA/cm^2^
Ni_78_Si_8_B_14_	41	42.8
Ni_70_Mo_20_Si_5_B_5_	72	11.0
Ni_58_Co_20_Si_10_B_12_	52	56.1
Ni_25_Co_50_Si_15_B_10_	45	67.4

### 4.2. Scanning Tunneling Spectroscopy Results

**Figure 6:**
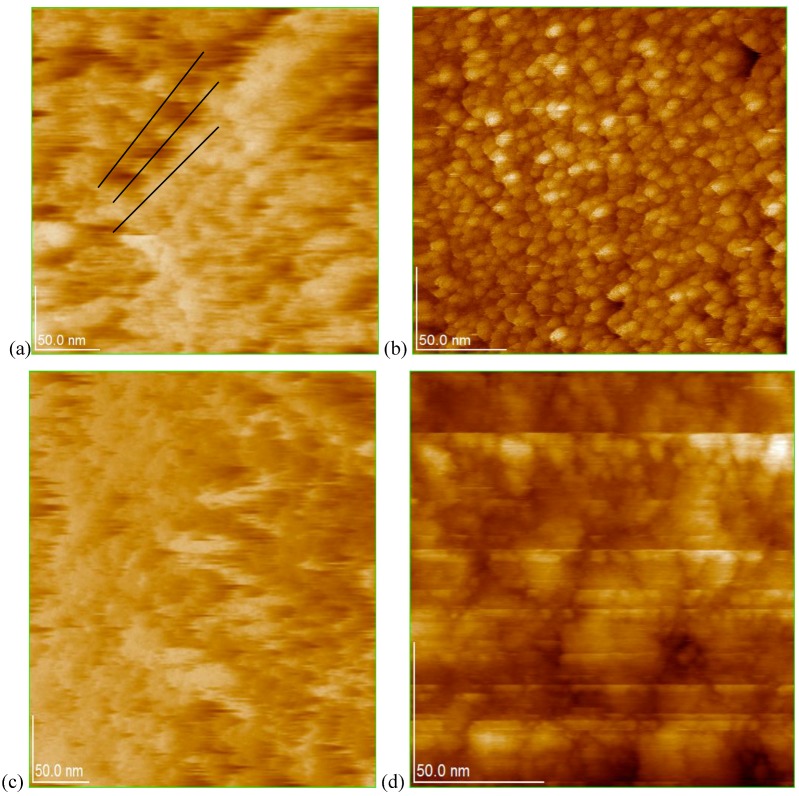
STM images of (a) Ni78Si8B14, (b) Ni70Mo20Si5B5, (c) Ni58Co20Si10B12, and (d) Ni25Co50Si15B10 after the OER. The images are obtained with a Pt-Ir tip at a bias voltage of 1.0 V and sample current 1.0 of nA in ambient air with atmospheric pressure.

**Figure 7:**
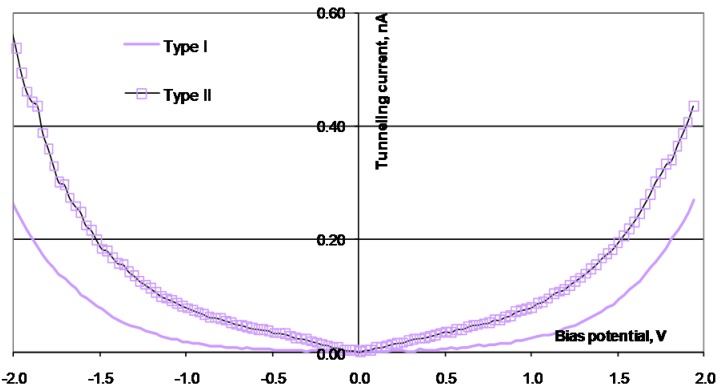
I(U) curves obtained from STS scans performed on the surface of Ni_78_Si_8_B_14_. The absolute value of the tunneling current is used.

**Figure 8:**
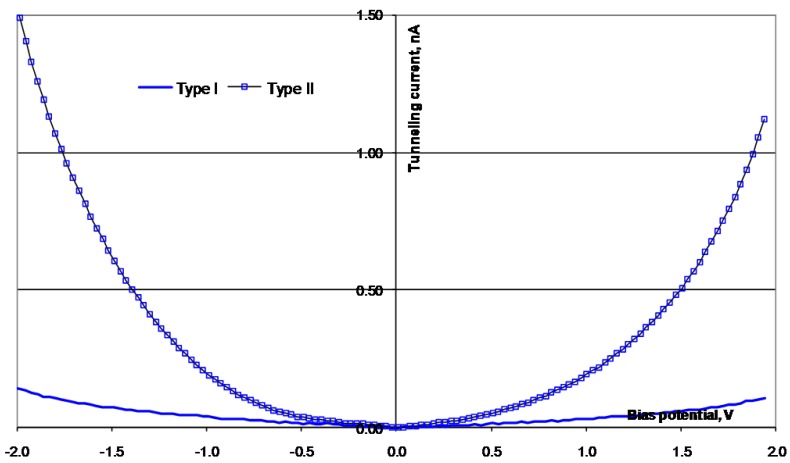
I(U) curves obtained from STS scans performed on the surface of Ni_70_Mo_20_Si_5_B_5_. The absolute value of the tunneling current is used.

**Figure 9:**
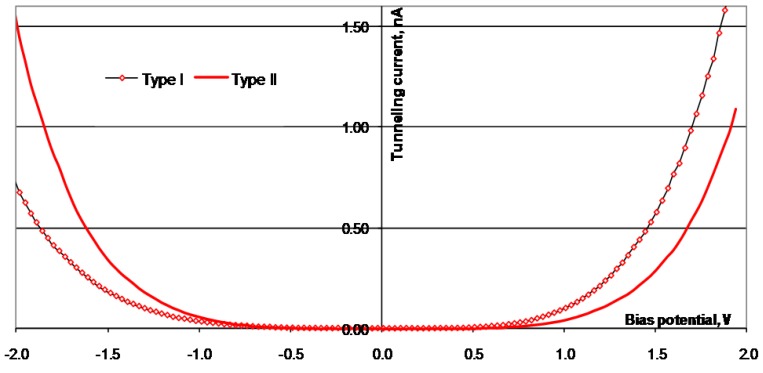
I(U) curves obtained from STS scans performed on the surface of Ni58Co20Si10B12. The absolute value of the tunneling current is used.

**Figure 10:**
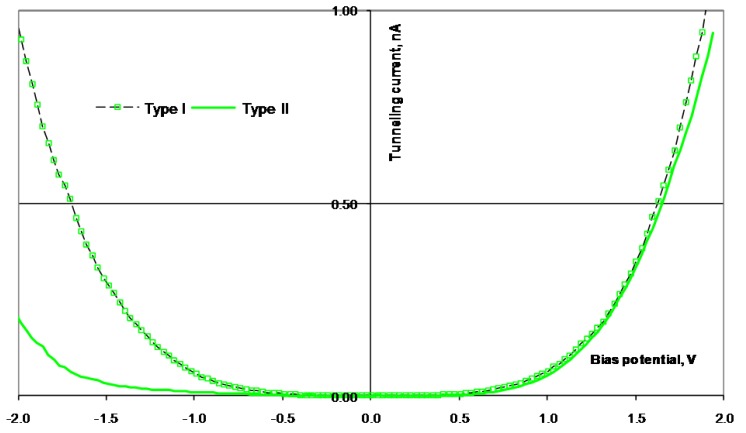
I(U) curves obtained from STS scans performed on the surface of Ni_25_Co_50_Si_15_B_10_. The absolute value of the tunneling current is used.

The surfaces of the alloys were investigated after the electrochemical tests were performed. Due to previous studies by Knutsen *et al.* [18,19], the alloying elements Si, B, Co, and Mo are expected to give changes in the electronic properties, both of the Ni atoms in the bulk, and also in the oxidized surface. The air-formed oxide layer on the surface of the alloys is expected to be NiO or Ni(OH)_2_. At the potential where the OER starts, the hydroxide is oxidized to NiOOH. An earlier investigation of the oxide/hydroxide layer on Ni_78_Si_8_B_14_ confirmed this assumption [18]. Examples of the surface regions used for the STS investigations are shown in the topographic images of Figure 6. Examples of the I(U) characteristics for the alloys are given in Figure 7, Figure 8, Figure 9 and Figure 10. The surfaces were found to be inhomogeneous with respect to the tunneling ability, and there sites that were found, which had different I(U) curves for all the alloys.

The STM images showed that even though the outermost surface of all the alloys consists of NiOOH, the structure of the surfaces was different. The image of Ni_78_Si_8_B_14_ revealed a surface with regular lines separated by a distance of approximately 20–25 nm, whilst the surface of Ni_70_Mo_20_Si_5_B_5_ was irregular and rougher, with several sites on the surface with either higher or lower concentrations of acceptors. The surface consists of clusters with a size of *ca.* 10 nm. Comparing the images of the alloys containing Co indicated that an increase in the content of Co gave a change in the uppermost surface. The image of Ni_25_Co_50_Si_15_B_10_ revealed an irregular, rough surface with larger clusters of *ca.* 20–25 nm, consisting of smaller clusters at a size of *ca.* 10 nm.

The STS investigations of the four alloys showed that the surfaces were inhomogeneous and there were sites with different tunneling properties on all the surfaces. All the surfaces revealed I(U) curves with different symmetry, both at different sites in the same alloy, and at sites on different alloys. For the surface of Ni_70_Mo_20_Si_5_B_5_, some of the sites had a higher tunneling current, in both directions, than other sites. There were no Coulomb blockage gaps and the curves were symmetric around the zero bias voltage. For the surface of Ni_78_Co_20_Si_10_B_12_, the I(U) curves indicated a Coulomb blockage gap around the zero bias voltage, and the I(U) curves were displaced compared to zero. Two distinctively different symmetry forms of the I(U) curves were found. All the I(U) curves found on the surface of Ni_25_Co_50_Si_15_B_10_ had been similarly shaped at positively biased voltages, whilst there were differences at negatively biased voltages. The I(U) curves revealed a Coulomb blockage gap, and the curves were displaced compared to the zero bias voltage, Type I in negative direction, and Type II in the positive direction. Also for the Ni_78_Si_8_B_14_ there were found two different kinds of I(U) characteristics—one which was symmetric around the zero bias voltage and with no Coulomb blockage gap, and one asymmetric with an Coulomb blockage gap. The charge ratio was calculated from the different I(U) characteristics and listed in Table 3. The sites where the I(U) scans were performed are expected to be clusters dominated by either Ni^2+^( r_c_ < 1) or O^2-^( r_c_ > 1).

**Table 3:** Charge ratio for Ni_78_Si_8_B_14_, Ni_70_Mo_20_Si_5_B_5_, Ni_58_Co_20_Si_10_B_12_, and Ni_25_Co_50_Si_15_B_10_ calculated from the STS curves in Figure 7, Figure 8, Figure 9 and Figure 10.

Alloy	Charge ratio, Clusters with Ni^2+^and/or Co^2+^,Co^3+^,Mo^4+^,Mo^6+^,H^+^	Charge ratio, Clusters with O^2-^
Ni_78_Si_8_B_14_	0.81 ± 0.05	1.22 ± 0.05
Ni_70_Mo_20_Si_5_B_5_	0.91 ± 0.07	1.04 ± 0.04
Ni_58_Co_20_Si_10_B_12_	0.61 ± 0.09	1.11 ± 0.01
Ni_25_Co_50_Si_15_B_10_	0.45 ± 0.12	1.17 ± 0.15

## 5. Discussion and Conclusions

The aim of this work was to find a relationship between the electrocatalytic properties of an electrode and its ability to tunnel electrons in an STM. The OER, in an alkaline electrolyte, was used as an indicative reaction for the electrocatalytic properties of different Ni alloys. The electrochemical tests all emphasized that the alloying elements had an influence on the electrocatalytic properties toward the OER. The electronic properties of the alloys depend both on the atomic type and on the amount of the alloying elements. According to the CVs (Figure 3) for all the alloys, the oxygen production started at potentials above the oxidation potentials for Ni^2+^/Ni^3+^, and in alkaline electrolyte, the uppermost surfaces of all the alloys will mainly be NiOOH. The surface sites will therefore be of either the negatively charged ion O^2-^ or the positively charged ions Ni^3+^and H^+^, in addition to traces of Ni^2+^, Co^2+^, Co^3+^, Mo^2+^, and Mo^4+^, depending on the alloys. Positively and negatively charged defects in the hydroxide structure may also exist.

According to Bockris and Khan [10] the first step in the OER, in alkaline electrolyte, will always be an adsorption of OH^-^, followed by the transfer of an electron from the adsorbate to the active site. This adsorption occurs on positively charged sites, and both the electronic properties and the amount of such sites play important roles in the electrocatalytic reaction. The Tafel slopes found for low overpotential (Table 2) indicated that the alloy containing Mo had a different rate determining step from the other alloys. According to several researchers [17,20,21,22,23], a Tafel slope at 40 mV/dec corresponds with a rate determining step that includes a transfer of electrons from adsorbed OH^-^ and O^-^ to the active sites. In contrast, a Tafel slope of 60–70 mV/dec, has a rate determining step that corresponds with other reactions and the number of active sites. By using the steady state current density, at an overpotential of 0.6 V, as an indication of the catalytic activity of the alloys (Figure 4 and Table 2), the alloys containing Co are found to be the most active toward the OER. That corresponds with the findings of Kreysa and Håkansson [15].

The STS investigations revealed the dependence of the tunneling current on the ramping bias voltage and that the alloying elements played a significant role in determining the size of the tunneling current. When using the same tip, the same bias voltage, at the interruption of the feedback controller, and the same scan rate for all the I(U) scans, differences, both in the size and the shape of the curves, can be retraced to differences in the surface investigated, and hence to differences in the charge ratio. Positively charged sites are supposed to result in r_c_ < 1, and negatively charged, in r_c_ > 1. Comparing the curves giving rise to r_c_ > 1 gives the opportunity compare the electron transferring ability to O^2- ^for the alloys. The largest value for r_c_ was found for Ni_78_Si_8_B_14_, indicating that both Mo and Co reduced the ability for clusters with O^2-^ to donate electrons to the tip.

Comparing the curves giving rise to r_c_ < 1 reveals differences in the electron transferring ability at positively charge sites, mainly in Ni^3+^. The alloys containing Co, (Ni_58_Co_20_Si_10_B_12_ and Ni_25_Co_50_Si_15_B_10_) were found to have smaller r_c_ values then Ni_78_Si_8_B_14_. The decrease indicates an increase in the ability to accept electrons from the tip. According to Martinez [24] and Nagai [25], Co atoms are transferring electrons to the Ni atoms in the alloys. At the overpotential of 0.6 V toward the OER, Co atoms are oxidized to Co^3+^ cations. When Co atoms are added to an NiSiB-alloy, an increase in the electrocatalytic activity towards the OER, a smaller charge ratio, and a smaller displacement of the Coulomb blockage gap are found. For the alloys Ni_58_Co_20_Si_10_B_12_ and Ni_25_Co_50_Si_15_B_10_, the Co atoms increase the creation of acceptors in the outermost surface of the produced NiOOH. This may be explained by the simultaneous formation NiCo_2_O_4_ from Ni and Co, with a change in the cation distribution [26]. According to the calculations of Nagai [25], the energy levels of Co 4s are located in the same energy region as that of Ni 4s, and there were no creations of mid-gap states. For both alloys, there were found Coulomb blockage gaps around zero bias voltage.

When Mo atoms are added to the NiSiB-alloy, the positively charged sites for the surface give rise to an increase in the charge ratio. These findings indicate that the alloying element Mo increase the surface’s ability to donate electrons to the tip. In the electrochemical tests, the addition of Mo resulted in a shift of the oxidation of Ni(II) to a lower potential and a decrease in the catalytic activity toward the OER. For Ni_70_Mo_20_Si_5_B_5_, the outermost surface may, in addition to Ni^3+^, Ni^2+^, and O^2-^, also contain some MoO_4_^2-^ ions. These ions are selective to adsorptions of cations, leading to a decrease in the amount of sites capable of adsorbing OH^-^ ions. The I(U) curves that were found from the alloy containing Mo had no Coulomb blockage gaps. The calculations of Nagai and Morisaki [25] showed that coupled levels of Mo 4d orbitals are located under the lowest level of Ni 4s, and that Mo 4d orbitals start to overlap with Ni 3d (located under the Fermi level), creating mid-gap states of Mo 4d. This finding can then explain both the lack of a Coulomb blockage gap and the creation of more LDOS under the Fermi level, giving an increase in the tunneling ability of electrons from the sample to the tip. The increased LDOS in the d-orbitals at the Fermi level, found by Martinez [24], may be the reason for the increased activity toward the HER [27] and a decreased activity toward the OER.

**Figure 11:**
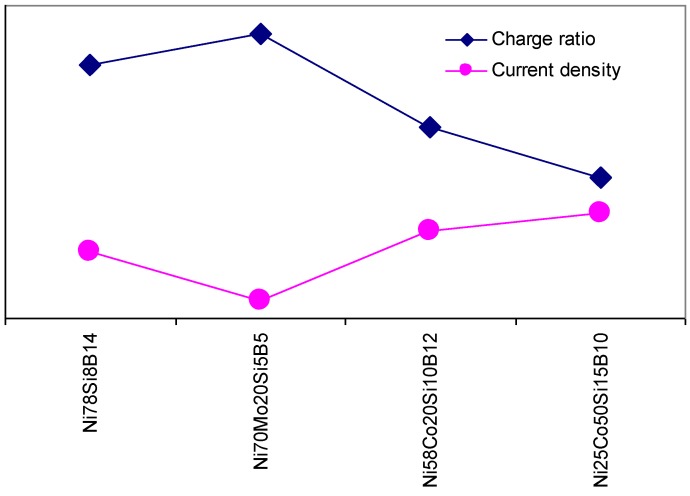
A comparison between the charge ratio at positively charged sites and the current density, at an overpotential of 0.6 V, within the Ni alloys investigated in this study. The scales are different for the two curves, and are here left out for simplicity.

Using the current density at an overpotential of 0.6 V toward the OER as an indication for the electrocatalytic activity of the alloys toward the OER, and the charge ratio as an indication of the tunneling ability of the surface, Figure 11 illustrates the correlation between the tunneling ability and the electrochemical activity toward the OER. In order to ease the comparison, different axes are used for the two sets of results. The curves show that the alloys with the highest electroactivity toward the OER have the lowest value for the charge ratio. Ni_25_Co_50_Si_15_B_10_ had the highest current density at the overpotential of 0.6 V. The Tafel slope was found to be 45 mV/dec. This slopes indicated that the reaction rate was determined by the electron transfer reaction [17,20,21]. When the rate determining step includes an electron transfer, it is reasonable to expect that the easier the sites accept electrons, the higher the OER activity is.

The results show a relationship between the ability of a surface site to tunnel electrons and the surface’s catalytic activity toward the OER. This will also indicate that to improve a catalyst for the OER with a low charge ratio, an increase in the number of active surface sites should be a priority. The use of STS in electrocatalysis testing may be an aid in the search for the optimal catalyst in many electrochemical processes. The size of the charge ratio and the Coulomb blockage gap of the I(U) curve found from the STS scans performed on the surface sites of an electrode may give an indication of the electrocatalytic activity of the electrode. However, the use must be done with care. In order to get the best comparison of different electrodes, the best results are found by using the same STM tip in all the experiments.
